# Interest in technology-based and traditional smoking cessation programs among adult smokers in Ankara, Turkey

**DOI:** 10.1186/1617-9625-9-10

**Published:** 2011-08-01

**Authors:** Michele L Ybarra, A Tülay Bağci Bosi, Nazmi Bilir, Jodi S Holtrop, Josephine Korchmaros, Salih Emri

**Affiliations:** 1 Internet Solutions for Kids, Inc., 555 N. El Camino Real #A347, San Clemente, CA 92672-6745, USA; 2 Department of Public Health, Hacettepe University School of Medicine, Ankara, 06100, Turkey; 3 Department of Family Medicine, Michigan State University, B101 Clinical Center, Michigan State University, East Lansing, MI 48824, USA; 4 Department of Chest Diseases, Hacettepe University School of Medicine, Ankara, 06100, Turkey

**Keywords:** smoking cessation, Turkey, middle income country, technology-based interventions

## Abstract

**Background:**

Little is known about the demand for smoking cessation services in settings with high smoking prevalence rates. Furthermore, acceptability of text messaging and Internet as delivery mechanisms for smoking cessation programs in non-developed countries is under-reported. Given the cost effectiveness of technology-based programs, these may be more feasible to roll out in settings with limited public health resources relative to in-person programs.

**Findings:**

148 adult smokers took part in a community-based survey in Ankara, Turkey. Two in five (43%) respondents reported typically smoking their first cigarette within 30 minutes of waking. Many participants expressed a desire to quit smoking: 27% reported seriously thinking about quitting in the next 30 days; 53% reported at least one quit attempt in the past year. Two in five smokers wanting to quit reported they were somewhat or extremely like to try a smoking cessation program if it were accessible via text messaging (45%) or online (43%).

**Conclusions:**

Opportunities for low-cost, high-reach, technology-based smoking cessation programs are under-utilized. Findings support the development and testing of these types of interventions for adult smokers in Turkey.

## 

Although smoking rates have decreased in high-income countries, rates in low- and middle-income countries seem to be resistant to declines [1]. This is especially true in Turkey, a middle income country [2] with a national population of almost 73 million people [3]. Turkey is 6^th ^in the world in smoking consumption [4] and is one of the top ten producers of tobacco [5]. An estimated 48% of Turkish men and 15% of Turkish women 15 years of age and older smoke cigarettes [5]. Indeed, a current smoker is found in seven out of 10 households [6]. Factors contributing to smoking prevalence are complex [7]. Because of the long lag time between onset of smoking and onset of disease however, cessation among current adult smokers is the path to reducing mortality within the next 25 years [8].

Implementation of effective smoking cessation programs in developing countries with high smoking prevalence rates such as Turkey is a major public health priority [5,9-11]. Even so, constrained public health resources make this difficult and literature documenting successful smoking interventions in Turkey is sparse. Demir and colleagues [12] report results of a pilot smoking cessation study conducted in an outpatient clinic where 40% of 118 patients using nicotine replacement therapy were quit at 3-months. Similarly, Emri and colleagues [13] report 46% of males and 37% of females were quit at 10 weeks using nicotine patches (n = 51) in their outpatient smoking cessation study. Yilmaz et al. [14] tested the effects of smoking intervention messages delivered to mothers during pediatric well-child visits on cessation (n = 363). Findings suggest that mothers who receive smoking intervention messages report significantly more smoking cessation or reduction behavior; this is especially true for mothers exposed to messages that focus on the health consequences for the child. No other cessation studies were found by the authors.

Smoking is culturally and socially acceptable in Turkey [15-17]. Nonetheless, there is suggestion of a strong demand to quit in some subpopulations. Unalacak and colleagues [18] report that 65% of coal workers who are smokers living in Zonguldak, Turkey want to quit and 60% reported making at least one quit attempt in the previous year. Given the lack of cessation research in Turkey, these data suggest there may be unmet demand for evidence-based smoking cessation programs among current smokers.

Technology, such as Internet and cell phone text messaging, represents a low-cost solution that overcomes many structural challenges and access issues that traditional programs have (e.g., lack of local services, transportation, competition for time otherwise spent on other activities, etc). Text messaging and Internet-based smoking cessation programs have reported promising short-term outcomes in developed countries [19,20]. Like many low and middle income countries, cell phone use has increased exponentially in Turkey. An estimated 53 million cell phones are owned in Turkey, making cell phones 2.7 times more common than land-line telephones [21]. With 27 million people online, Turkey ranks 15^th ^in the world in the gross number of Internet users [21]. The wide use of cell phones and Internet suggests that these technologies may be a feasible delivery method for urgently needed smoking cessation programs in the country.

Demand for smoking cessation services in Turkey is largely unknown. Furthermore, whether people who want to quit are willing to use smoking cessation services delivered through new technologies needs to be investigated. To address these gaps in knowledge, the current study aims to: 1) measure interest in smoking cessation services among current smokers; and 2) examine interest in services that are technology-based specifically, while comparing these rates to interest in traditional services.

## Methods

A community-based survey of adults was conducted in Ankara, Turkey between April and July, 2008. As the capital of Turkey, Ankara is the second largest city in Turkey after Istanbul. Ankara is in the heart of the Anatolian Peninsula. As the bridge way between Asia and Europe, this peninsula is a main trading route for tobacco [22]. It is estimated that at least one smoker resides in 70% of the houses in the South-eastern Anatolian Region, which is similar to the country as a whole [6]. Forty-one percent of Ankara adults are smokers, ranked third in prevalence behind Istanbul (44%) and Izmir (44%) [23]. Ankara is, thus, characteristic of many cities in the Middle East with a high smoking prevalence.

The protocol was reviewed and approved by Western IRB in the United States and by Hacettepe University in Ankara, Turkey. Eligibility criteria included: being over 18 years of age, currently smoking daily, owning a cell phone and using text messaging in the past year, and informed consent. Ineligibility criteria included having a serious health condition (i.e., emphysema, heart disease, lung disease).

A research assistant went to government buildings and solicited those smoking outside to take part. Flyers also were posted in the common areas at Hacettepe University. Respondents were told that "we are designing a program to help adults quit smoking and we need your input".

To query the acceptability of technology-based smoking cessation programs, respondents were asked: "If there was a program designed to help you stop smoking that used the following medium to deliver the information to you, how likely would you be to try it?" Six mediums were asked: In person (group), Telephone (one on one), E-mail, Text (SMS) messages, Multimedia (MMS) messages, and web site. Participants answered on a 5-point scale [1 (extremely unlikely) - 5 (extremely likely)]. Respondents provided general information about their demographic characteristics and technology use. Perceptions and norms related to smoking were queried using items developed by Nierkens and colleagues (e.g., "Smoking is a waste of money") [24].

## Results

Among the 165 adults who were identified as eligible, 152 adults completed the self-report survey (response rate = 91%). Among interested adults, reasons for ineligibility included: not living in Ankara, having a smoking-related chronic disease, and not seriously thinking about quitting in the next 30 days. Four respondents were deemed ineligible during data cleaning; three because they reported not owning or using a cell phone, and one because of the report of no cigarette smoking in the past seven days. Consequently, the final sample size was 148.

Characteristics of the 148 respondents are shown in Table 1. Respondents were similar to the general population in Turkey, albeit with a slightly higher monthly income (1,250 - 1,999 Turkish lira) than average (1,103 Turkish lira) [25]. Two in five respondents (43%) reported that they usually smoked their first cigarette within 30 minutes of waking. An important minority of respondents (27%) reported a desire to quit in the next 30 days.

**Table 1:** Characteristics of survey respondents (n = 148)

Personal Characteristics	% (n)
Demographic characteristics	
Age (M: SD) range: 19-63	37.8 (10.0)
Female	44% (65)
Married	64% (94)
Household income (per month)	
1249 ytl and less	26% (39)
1250 - 1999 ytl	28% (41)
2000 - 4000 ytl	27% (40)
More than 4000 ytl	17% (25)
Missing/don't know	2% (3)
Technology Characteristics	
Send/receive text messages everyday/almost every day	43% (63)
Use email everyday/almost everyday	38% (56)
Use Internet (other than email) everyday/almost everyday	49% (73)
Health characteristics	
Asthma	11% (16)
Respiratory allergies	10% (15)
Smoking characteristics	
Someone else in the household currently smoking	8% (12)
Family member currently smoking	80% (112)
Family member has quit smoking	59% (88)
Current smoker of narghile (water pipe)	5% (7)
First cigarettes of the day within 30 minutes of waking	43% (63)
Average number of cigarettes in a day	
1-5 cigarettes	9% (14)
6-15 cigarettes	43% (63)
16-25 cigarettes	38% (56)
26-40 cigarettes	10% (14)
40 or more cigarettes per day	< 1% (1)
Seriously thinking about quitting	
In the next 30 days	27% (40)
In the next 6 months	14% (20)
Sometime	39% (58)
Never	18% (26)
Missing	3% (4)
Average number of quit attempts for 24 hours or more in the past year (M:SD)	1.2 (1.5)

Just over half (53%) of respondents reported a past-year quit attempt. Although quit attempts were commonly reported, few attempters (23%) reported using a cessation aid.

As shown in Table 2, smoking was generally viewed negatively. Only 28% of respondents indicated that they agreed or strongly agreed that smoking was "normal" and only 12% agreed that it was "cool". Conversely, 77% agreed that smoking was a waste of money and 94% agreed that it was bad for one's health.

**Table 2:** Norms for smoking (n = 148)

Perceptions about smoking (agree/strongly agree that...)	
Negative attitudes towards smoking	
Smoking is annoying for people around the smoker	94% (139)
Smokers are not setting a good example for their children	90% (133)
Smoking is a waste of money	77% (114)
Smoking has a negative health impact on self and others	
Smoking is bad for the health of people around the smoker	94% (139)
Smoking is bad for the smoker's health	94% (139)
Smokers have a higher chance of getting lung diseases	90% (133)
Smokers have a higher chance of getting heart diseases	90% (133)
Smokers cough more	83% (123)
Positive attitudes towards smoking	
Smoking is normal	28% (41)
Smoking is not as bad for you as they make it sound	18% (27)
Smoking is cool	12% (18)

All respondents had access to each type of smoking cessation program delivery medium asked about--in-person, telephone, email, cell phone-based text messaging and multimedia messaging, and web site. Seven in ten (69%) respondents said that they were somewhat or extremely likely to try at least one of the six quitting aids queried if they were trying to quit. Among those seriously thinking about quitting in the next 30 days (27%, n = 40), many indicated they would be somewhat or extremely likely to try a program if it were available in-person (63%), via telephone (53%), on email (51%), via text messaging (45%), or online (43%) with some somewhat or extremely likely to try a program if it were available via multi-media text messaging (20%; see Table 3). Responses generally were similar for smokers who were not seriously thinking about quitting. Of exception, a greater proportion of the smokers seriously thinking about quitting (51%) than those not seriously thinking about quitting (28%) said they were somewhat or extremely likely to access a smoking cessation service via email (p = 0.04).

**Table 3:** Likelihood of accessing a smoking cessation program based upon delivery mechanism

	Seriously thinking about quitting in the next 30 days (n = 40)	Not seriously thinking about quitting in the next 30 days (n = 108)		
			
Mode of intervention delivery	% (n)	% (n)	Statistical comparison	p-value
In-person (group)			χ^2^(5) = 7.2	0.21
Extremely Likely	33% (13)	15% (16)		
Somewhat Likely	30% (12)	31% (33)		
Neither Likely nor unlikely	18% (7)	19% (21)		
Somewhat Unlikely	10% (4)	18% (19)		
Extremely Unlikely	10% (4)	16% (17)		
Decline to answer	0% (0)	2% (2)		
Phone			χ^2 ^(5) = 6.9	0.23
Extremely Likely	25% (10)	13% (14)		
Somewhat Likely	28% (11)	22% (24)		
Neither Likely nor unlikely	20% (8)	15% (16)		
Somewhat Unlikely	15% (6)	30% (32)		
Extremely Unlikely	10% (4)	17% (18)		
Decline to answer	3% (1)	4% (4)		
Email			χ^2 ^(5) = 11.6	0.04
Extremely Likely	28% (11)	9% (10)		
Somewhat Likely	23% (9)	19% (20)		
Neither Likely nor unlikely	13% (5)	8% (9)		
Somewhat Unlikely	20% (8)	38% (41)		
Extremely Unlikely	15% (6)	19% (21)		
Decline to answer	3% (1)	6% (7)		
Text messaging			χ^2 ^(5) = 3.2	0.66
Extremely Likely	15% (6)	9% (10)		
Somewhat Likely	30% (12)	22% (24)		
Neither Likely nor unlikely	10% (4)	10% (11)		
Somewhat Unlikely	23% (9)	34% (37)		
Extremely Unlikely	20% (8)	19% (21)		
Decline to answer	3% (1)	5% (5)		
Multi-media text messaging			χ^2 ^(5) = 2.6	0.76
Extremely Likely	5% (2)	6% (6)		
Somewhat Likely	15% (6)	10% (11)		
Neither Likely nor unlikely	5% (2)	7% (8)		
Somewhat Unlikely	35% (14)	45% (49)		
Extremely Unlikely	33% (13)	23% (25)		
Decline to answer	8% (3)	8% (9)		
Internet			χ^2 ^(5) = 8.3	0.14
Extremely Likely	18% (7)	7% (8)		
Somewhat Likely	25% (10)	18% (19)		
Neither Likely nor unlikely	8% (3)	8% (9)		
Somewhat Unlikely	28% (11)	37% (40)		
Extremely Unlikely	23% (9)	20% (22)		
Decline to answer	0% (0)	9% (10)		

## Discussion

Despite the high smoking prevalence in Ankara, [23] a city that is characteristic of many cities in middle income countries with high smoking prevalence, [26] many respondents report a desire to quit smoking. Furthermore, contrary to previous reports of social acceptability of smoking, [24] smoking is not viewed by the smokers in the current survey as a positive behavior. This finding may be because the population surveyed is more educated than the general public. Nonetheless, it seems that there is a population of smokers who view smoking negatively and are trying to quit, albeit unsuccessfully.

Findings suggest that there is interest in cessation services among smokers. Among the most likely population to access services - smokers seriously thinking about quitting in the next 30 days - the majority said they would likely use cessation services if they were available. This included both traditional and technology-based options. Traditional services such as in-person and telephone counseling engendered more interest than technology-based services among survey respondents. These services require relatively more resources however, and have limited accessibility. Indeed, Turkey's Ministry of Health has developed and is implementing a free quit line [personal communication, Emri, January, 2011]. Anecdotal accounts suggest that the infrastructure is insufficient to meet demand; some people are waiting over 12 minutes on hold before speaking with a health educator. Opportunities to integrate higher cost efforts (e.g., quit lines) with more cost effective methods (e.g., text messaging) might be considered by public health officials. For example, text messages could reinforce information conveyed by the telephone counselor; or smokers could use the communication tool to reach out to the counselor when in crisis. This coupling of communication methods could increase the efficiency and, therefore, capacity of the telephone quit line.

Two in five respondents seriously thinking about quitting in the next 30 days expressed interest in text messaging and Internet-based cessation services. Reviews suggest that these delivery modes can be associated with behavior change at least in the short term [19,20]. Interventions have been reported in New Zealand, the United States, England, and Australia. Data suggest exponential technology growth in Turkey, [21] with similar usage increases noted in other countries in the Middle East and Asia [27,28] that also have high smoking prevalence. Efforts to develop and test the efficacy of text messaging and Internet-based smoking cessation services in these countries are warranted.

Multimedia text messages had the lowest rate of interest among smokers. This may be because of the associated cost to both own an MMS-capable phone, as well as to send and receive these types of messages. This is in contrast to SMS, which is free to receive. Not all technology is equal, nor is access homogenous. Researchers are encouraged to consider the target population and where they "are". While MMS may provide more flexibility in programming, it reaches fewer people. If the lure of technology is its reach, then we diminish its impact by choosing less ubiquitous platforms. This said, researchers also must try to anticipate trends and develop programs for where populations may "be" in the future given the long lag time between development and implementation.

Study limitations include self-selection bias of the convenience sample, and the small sample size. Because of recruiting respondents near government buildings and Hacettepe University, our sample might be more educated, which may be related to increased interest in technology-based interventions compared to the general smoking population. This possibility, however, is offset by the fact that participants were told that "we are designing a program to help adults quit smoking"; participants did not self-select based upon interest in technology-based programs per se. It also should be noted that intention and interest in smoking cessation services (or lack thereof) do not equal participation (or lack of participation) in these services.

## Conclusion

Findings suggest that some adult smokers in Ankara may be interested in utilizing treatments to aid in cessation, including low-cost, high-reach technology-based applications such as text messaging, email, and web sites. Public health efforts should be focused on increasing the availability of and access to evidenced-based smoking cessation programs, including those that harness new technologies. As an example, these data will be used by the authors to inform the development of a text messaging-based smoking cessation program for adult smokers in Ankara.

## Competing interests

The authors declare that they have no competing interests.

## Authors' contributions

MLY, ATBB, and SE made substantial contribution to the survey conception and design. ATBB and SE made substantial contribution to the acquisition of data. MLY analyzed the data and drafted the article. All authors (MLY, ATBB, SE, NB, JSH, and JK) made substantial contribution to the interpretation of data and revised it critically for important intellectual content. All authors read and approved the final manuscript.

## Funding

The project described was supported by Award Number R01TW007918 from the Fogerty International Center. The content is solely the responsibility of the authors and does not necessarily represent the official views of the Fogerty International Center or the National Institutes of Health.
